# Structure of a GH51 α-l-arabinofuranosidase from *Meripilus giganteus*: conserved substrate recognition from bacteria to fungi

**DOI:** 10.1107/S205979832001253X

**Published:** 2020-10-16

**Authors:** Nicholas G. S. McGregor, Johan P. Turkenburg, Kristian B. R. Mørkeberg Krogh, Jens Erik Nielsen, Marta Artola, Keith A. Stubbs, Herman S. Overkleeft, Gideon J. Davies

**Affiliations:** a York Structural Biology Laboratory, University of York, Heslington, York YO10 5DD, United Kingdom; b Protein Biochemistry and Stability, Novozymes A/S, Krogshøjvej 36, 2880 Bagsvaerd, Denmark; c Leiden Institute of Chemistry, Leiden University, Einsteinweg 55, 2300 RA Leiden, The Netherlands; d School of Molecular Sciences, The University of Western Australia, 35 Stirling Highway, Crawley, Western Australia 6009, Australia

**Keywords:** glycoside hydrolases, arabinofuranosidases, cyclophellitol, iminosugar, sulfur SAD

## Abstract

α-l-Arabinofuranosidases from glycoside hydrolase family 51 use a stereochemically retaining hydrolytic mechanism to liberate nonreducing terminal α-l-arabinofuranose residues from plant polysaccharides such as arabinoxylan and arabinan. To date, more than ten fungal GH51 α-l-arabinofuranosidases have been functionally characterized, yet no structure of a fungal GH51 enzyme has been solved. In contrast, seven bacterial GH51 enzyme structures, with low sequence similarity to the fungal GH51 enzymes, have been determined. Here, the crystallization and structural characterization of *Mg*GH51, an industrially relevant GH51 α-l-arabinofuranosidase cloned from *Meripilus giganteus*, are reported. Three crystal forms were grown in different crystallization conditions. The unliganded structure was solved using sulfur SAD data collected from a single crystal using the I23 *in vacuo* diffraction beamline at Diamond Light Source. Crystal soaks with arabinose, 1,4-dideoxy-1,4-imino-l-arabinitol and two cyclophellitol-derived arabinose mimics reveal a conserved catalytic site and conformational itinerary between fungal and bacterial GH51 α-l-arabino­furanosidases.

## Abstract 2

The structure of a fungal α-l-arabinofuranosidase has been determined using sulfur SAD data collected *in vacuo* using beamline I23 at Diamond Light Source. Complexes with l-arabinose, an iminosugar and two covalent inhibitors confirm the identity of the catalytic nucleophile and provide a detailed map of enzyme–substrate interactions that are conserved between bacteria and fungi.

## 1. Introduction   

α-l-Arabinofuranosidase activity is an important part of catabolic pathways which facilitate the saccharification of hemicellulosic and pectinaceous polysaccharides such as xyloglucan (Hemsworth *et al.*, 2016 ▸), arabinoxylan (Rogowski *et al.*, 2015 ▸) and arabinan (Matsuo *et al.*, 2000 ▸). These potential substrates contain a variety of arabinosyl linkage types, including α(1,2) and α(1,3) linkages to xylopyranose (Izydorczyk & Biliaderis, 1994 ▸; York *et al.*, 1996 ▸), and α(1,2), α(1,3) and α(1,5) linkages to arabinofuranose (Wefers *et al.*, 2018 ▸). Exo-α-l-arabinofuranosidases, such as those found in glycoside hydrolase (GH) families 43, 51, 54 and 62 (Lombard *et al.*, 2014 ▸), often display a broad substrate specificity, facilitating the degradation of multiple polysaccharide substrates (Beylot *et al.*, 2001 ▸; Sakamoto *et al.*, 2013 ▸).

The saccharification of arabinoxylan, a major component of non-starch biomass derived from grasses (McNeil *et al.*, 1975 ▸), by fungi can be limited by incomplete side-chain removal, reducing the overall efficiency with which the biomass can be broken down and converted (Gilbert, 2010 ▸). The activities of both GH51 and GH54 α-l-arabinofuranosidases are known to be hindered by steric crowding around the α-l-arabino­furanose residue (Koutaniemi & Tenkanen, 2016 ▸). Specifically, an industrial fungal GH51 α-l-arabinofuranosidase from *Meripilus giganteus* (*Mg*GH51) requires a synergistic GH43 enzyme from *Humicola insolens* (AXHd3) to completely debranch arabinoxylan (Sørensen *et al.*, 2006 ▸). *Mg*GH51 efficiently removes α-l-arabinofuranose residues from the 2 or 3 positions of monosubstituted xylose residues, while AXHd3 cleaves α-l-arabinofuranose residues from xylose residues which are substituted at both the C2 and C3 hydroxyl groups. Structural studies of AXHd3 have shown that it contains a second shallow pocket adjacent to the active-site cleft which accommodates the additional C2-linked α-l-arabinofuranose residue (McKee *et al.*, 2012 ▸), an active-site structure distinct from those of *Bacillus subtilis* AXH-m2,3 (Vandermarliere *et al.*, 2009 ▸) and *Cellvibrio japonicus* Arb43A (Nurizzo *et al.*, 2002 ▸), which display similar activities.

The structure of the *Mg*GH51 active site, and thus the basis of its specificity and activity, remains unknown. To date, a variety of GH51 enzymes displaying similar properties to *Mg*GH51 have been identified, including enzymes from *Aspergillus* sp. (Koseki *et al.*, 2003 ▸; Bauer *et al.*, 2006 ▸; Koutaniemi & Tenkanen, 2016 ▸), *Penicillium chrysogenum* (Sakamoto & Kawasaki, 2003 ▸) and *Talaromyces purpureogenus* (Fritz *et al.*, 2008 ▸), suggesting that the niche of GH51 is conserved across the fungal kingdom, yet no structure of a fungal GH51 α-l-arabinofuranosidase has been solved. Owing to the low sequence similarity between bacterial and fungal GH51 enzymes, it is not possible to generate a reliable sequence alignment or homology model for any characterized fungal GH51 enzyme, thus limiting our understanding of this enzyme class.

Structural investigations of the catalytic mechanism of bacterial GH51 α-l-arabinofuranosidases have primarily leveraged site-directed mutagenesis. A complex between an acid/base mutant of AbfA from *Geobacillus stearothermo­philus* and two substrates, 4-nitrophenyl α-l-arabinofuranoside and Ara-α(1,3)-Xyl, revealed a ^4^ *E* ring conformation in the Michaelis complex, priming the substrate for nucleophilic attack (Hövel *et al.*, 2003 ▸). Similar mutagenesis and crystallographic analysis of an α-l-arabinofuranosidases from *Clostridium thermocellum* (*Ct*Araf51A) revealed that loose specificity towards arabinan and arabinoxylan was facilitated by a +1 subsite which interacted primarily through hydrophobic stacking interactions and not more geometrically constrained hydrogen-bonding interactions (Taylor *et al.*, 2006 ▸).

**Figure 1:**
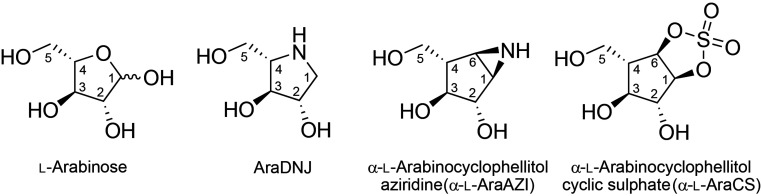
Structures of the ligands used in this study.

Chemical biology tools provide a more facile approach to the study of fungal enzymes, which require considerably more work to genetically engineer. For example, reversible inhibitors such as 1,4-dideoxy-1,4-imino-l-arabinitol (AraDNJ) have been applied to the study of GH62 α-l-arabino­furanosidases, binding in a Michaelis complex-like ^4^ *E* conformation and facilitating the identification of the catalytic water molecule of an inverting α-l-arabinofuranosidase (Moroz *et al.*, 2018 ▸). Irreversible inhibitors, such as α-l-arabinocyclophellitol aziridine (α-l-AraAZI) and α-l-arabinocyclophellitol cyclic sulfate (α-l-AraCS), have recently been shown to label the active sites of both GH51 and GH54 α-l-arabinofuranosidases, often mimicking the expected ^2^ *E* conformation of the glycosyl-enzyme intermediate (McGregor *et al.*, 2020 ▸). Using this chemical biology approach, we aimed to determine the structure of a fungal GH51 enzyme, identify its key catalytic residues and compare the catalytic sites and mechanisms of fungal and bacterial GH51 enzymes. To this end, we have crystallized *Mg*GH51 and solved its structure in the unliganded state and in complex with l-arabinose, AraDNJ, α-l-AraAZI and α-l-AraCS (Fig. 1 ▸). Owing to the challenges faced in solving the phase problem for this enzyme (see below), the I23 *in vacuo* beamline (Wagner *et al.*, 2016 ▸) was leveraged to solve the structure using sulfur SAD (Rose *et al.*, 2015 ▸), a technique that has been made tremendously more accessible by this new beamline.

## 2. Materials and methods   

Reagents were purchased from Sigma Millipore unless otherwise stated. The synthesis of AraDNJ was carried out according to literature procedures (Jones *et al.*, 1985 ▸; Naleway *et al.*, 1988 ▸), as were the syntheses of the cyclophellitol-derived inhibitors (McGregor *et al.*, 2020 ▸).

### 2.1. Enzyme production and purification   

Culture broth containing secreted *Mg*GH51 (Table 1 ▸) was produced as described by Sørensen *et al.* (2006 ▸). Filtrated broth was applied onto a Sephadex G-25 medium (GE Healthcare, Piscataway, New Jersey, USA) column equilibrated with 25 m*M* sodium acetate pH 4 and applied onto a SOURCE 15S column (GE Healthcare, Piscataway, New Jersey, USA) equilibrated with the same buffer. Bound proteins were eluted with a linear gradient from 0 to 1000 m*M* sodium chloride over ten column volumes. Fractions were collected and analysed by SDS–PAGE. *Mg*GH51-bearing fractions were pooled and the pH was adjusted to 5.5.

### 2.2. Substrate-hydrolysis kinetic measurements   

3^2^-α-l-Arabinofuranosyl-xylobiose (AX2), α(1,5)-linked arabinotriose (A3) and α(1,5)-linked arabinopentaose (A5) were purchased from Megazyme International (Bray, Ireland). Hydrolytic kinetics were measured essentially as described in McGregor *et al.* (2017 ▸). Briefly, reactions were set up at 40°C in 50 m*M* sodium acetate buffer pH 4 containing 5 ng ml^−1^ *Mg*GH51 and between 0.02 and 2.5 m*M* substrate. Reactions were stopped by the addition of ammonium hydroxide to give a final pH of 10.1 and free l-arabinose was quantified by the separation of 5 µl of the resulting solution on a CarboPac PA20 column at a flow rate of 0.5 ml min^−1^. The separation program was a 5 min isocratic flow of 50 m*M* sodium hydroxide, followed by a 5 min gradient to 50 m*M* sodium hydroxide, 100 m*M* sodium acetate, followed by a 3 min equilibration under the initial conditions. l-Arabinose was quantified relative to a calibration curve ranging from 2 to 250 µ*M*.

### 2.3. Crystallization and complex preparation   

Crystallization screening was carried out by sitting-drop vapour diffusion. Droplets containing 150 nl reservoir solution (RS) and either 150 or 300 nl protein solution (PS) were dispensed into 96-well MRC 2-well crystallization microplates (Swissci, Switzerland) and were equilibrated against 50 µl RS. Duplicate plates were stored at 6 or 20°C. *Mg*GH51 crystals grew under two different conditions (Table 2 ▸). Trays set up at 20°C with 10 mg ml^−1^ *Mg*GH51 in 10 m*M* sodium acetate buffer pH 5.5 with 100 m*M* NaCl yielded radial plate clusters (type 1) with 2:1 PS:RS, where the RS consisted of 0.1 *M* MES buffer pH 6, 0.2 *M* NaCl, 20% PEG 6000. A mixture of isolated rectangular and square plates with rounded edges (type 2) and rectangular prisms (type 3) grew from 2:1 PS:RS at 20°C, where the RS consisted of 2.2 *M* ammonium sulfate, 20% glycerol. Optimized type 1 crystals (Supplementary Fig. S1*a*) grew from 20% PEG 3350, 0.1 *M* bis-Tris–HCl pH 6.5, 0.2 *M* sodium nitrate in 1–3 days and optimized type 2 crystals (Supplementary Fig. S1*b*) grew from 2.4 *M* ammonium sulfate, 0.1 *M* sodium acetate pH 6.0, 20% glycerol in 2–7 days. High-quality type 3 crystals (Supplementary Fig. S1*c*) grew more commonly from 1.8 *M* ammonium sulfate, 0.1 *M* sodium acetate pH 5.0–6.0, 35% glycerol in 5–14 days. Mixtures of types 2 and 3 were rare; the growth of type 3 crystals appeared to correlate with a lack of type 2 crystals.

Crystals were cryocooled in liquid nitrogen without additional cryoprotection. All complexes were generated from type 1 crystals owing to competition for active-site binding from glycerol and the occlusion of the active site by a neighbouring molecule in type 2 and type 3 crystals. To generate the l-arabinose and AraDNJ complexes, crystals were soaked in RS containing 100 m*M* l-arabinose or 10 m*M* AraDNJ for 15–30 min prior to cryocooling. To generate complexes with cyclophellitol derivatives, 200 nl of 1 m*M* inhibitor (approximately one equivalent) was added directly to a 1500 nl crystal-containing droplet. This was left to stand at room temperature for 1 h prior to crystal cryocooling. Bromide-soaked crystals were prepared following literature methods (Pike *et al.*, 2016 ▸). Briefly, type 1 and type 2 crystals were soaked in reservoir solution prepared with 1 *M* sodium bromide for 2–10 min prior to cryocooling. Oligosaccharide soaks were also performed with 10 m*M* xylopentaose or 10 m*M* cellohexaose for 1 h prior to cryocooling.

### 2.4. Data collection and processing   

All data were collected and processed at Diamond Light Source (DLS), Harwell, UK. Initial native data sets for type 1 and type 2 crystals were collected on beamline I03 at a wavelength of 0.9763 Å to 1.30 and 1.33 Å resolution, respectively (Table 3 ▸). Three bromine SAD data sets were collected to 1.8–1.9 Å resolution from separate sodium bromide-soaked type 1 crystals (type 2 crystals did not tolerate NaBr soaking) on beamline I04-1 at a wavelength of 0.9159 Å (data not shown). Five sulfur SAD data sets were collected to 1.8 Å resolution (2θ-limited) from a single type 3 crystal at κ angles ranging from 0° to −25° using a wavelength of 2.75 Å on beamline I23 (Table 3 ▸). Two high-resolution data sets were collected to 1.2 Å resolution (crystal-limited) from a single type 3 crystal at κ angles of 0° and −20° using a wavelength of 1.375 Å on beamline I23. Single data sets were collected from arabinose-, α-l-AraAZI- and α-l-AraCS-soaked type 1 crystals at a wavelength of 0.9119 Å on beamline I04-1 (Table 3 ▸). Three data sets were collected from two AraDNJ-soaked type 1 crystals at a wavelength of 0.9795 Å on beamline I04 and were merged (Table 3 ▸). Unless otherwise indicated, the data sets were processed using the *xia*2 pipeline (Winter *et al.*, 2013 ▸) at DLS with *DIALS* (Winter *et al.*, 2018 ▸). All other calculations were carried out using *CCP*4 (Winn *et al.*, 2011 ▸) and figures were prepared using *PyMOL* (Schrödinger).

### 2.5. Structure solution and refinement   

Using the native data sets collected from type 1 and type 2 crystals, attempts were made to solve the structure of *Mg*GH51 by molecular replacement. The type 1 data set was indexed in space group *P*2_1_2_1_2_1_ and the type 2 data set was indexed in space group *C*222_1_. Molecular replacement with *Phaser* (McCoy *et al.*, 2007 ▸) using native models and *CHAINSAW*-modified (Stein, 2008 ▸) models generated from chain *A* of *Thermotoga maritima* GH51 (*Tm*GH51; PDB entry 3ug3; Im *et al.*, 2012 ▸) or *Thermobacillus xylanilyticus* GH51 (*Tx*GH51; PDB entry 2vrk; Paës *et al.*, 2008 ▸) failed to find any significant solutions. Automated molecular replacement using *MrBUMP* (Keegan & Winn, 2008 ▸) also failed to find any solutions. *Ab initio* phasing with *Fragon* (Jenkins, 2018 ▸) was also attempted, but failed, likely owing to the relatively small fraction of the total scattering which could be accounted for by fragments. Attempts were made to solve the bromine SAD data sets using *CRANK*2 (Pannu *et al.*, 2011 ▸), although a substructure that provided sufficient phasing power could not be determined.

Sulfur SAD phases were determined experimentally with the *CRANK*2 pipeline using the first two of the five data sets collected. The collection and merging of all of the data sets from different κ orientations was not necessary for phasing, but improved the overall data completeness and quality for model building and refinement. Substructure determination identified a collection of 16 peaks which could be modelled as S atoms with occupancy values of at least 0.1. These included six peaks which could be modelled as S atoms with full occupancies of 1 (giving anomalous map peak heights between 32σ and 54σ) and ten weaker peaks which could be modelled as partial occupancy S atoms (five of which gave anomalous map peak heights of at least 10σ). Initial automated chain tracing was performed using *ARP*/*wARP*. Following the correction of two *cis*-proline residues and merging of the three traced chains into one, the structure was refined by successive rounds of manual model building and refinement using *Coot* (Emsley *et al.*, 2010 ▸) and *REFMAC*5 (Murshudov *et al.*, 2011 ▸) within the *CCP*4 suite (Winn *et al.*, 2011 ▸). Sulfate molecules and chloride ions were modelled with positions and occupancies derived from anomalous refinement. Glycan structures, glycerol molecules and acetate molecules were modelled manually, and water molecules were then added automatically with manual adjustment. All other data sets were phased by *MOLREP* (Vagin & Teplyakov, 2010 ▸) using the sulfur SAD model prior to manual model adjustment and ligand modelling. Final refinement statistics for each structure can be found in Table 4 ▸.

## 3. Results and discussion   

### 3.1. Protein crystallization and structure determination   

*Mg*GH51 was very amenable to crystallization. Crystals of *Mg*GH51 reliably grew from two different conditions with relatively broad ranges of reservoir-solution composition. Three separate types of crystals were identified. One, which was obtained when PEG was used as the major precipitant (Supplementary Fig. S1*a*), grew optimally from bis-Tris pH 6.5 with 0.2 *M* sodium nitrate and diffracted to ∼1.3 Å resolution in space group *P*2_1_2_1_2_1_. Similar conditions yielded crystals of varying size and nucleation density with 0.2 *M* sodium sulfate, sodium acetate, sodium formate, sodium malonate or sodium tartrate in place of sodium nitrate and variable pH values from 6.5 to 8 with bis-Tris or Tris buffer. Crystals grew more rapidly (in some cases overnight) with reservoir-solution PEG concentrations of as high at 25%, but eventually fractured and dissolved as the droplet continued to equilibrate against the reservoir solution. Lower salt concentrations gave precipitation, and salt concentrations above 0.4 *M* strongly inhibited crystal nucleation. The major crystal pathologies were high anisotropy, splitting and high mosaicity, necessitating careful crystal selection and testing prior to diffraction.

Two other crystal types were observed in a narrower set of conditions with reservoir solutions composed of ammonium sulfate and glycerol with sodium acetate buffer. Type 2 crystals grew as a mixture of square or rectangular plates with rounded edges (Supplementary Fig. S1*b*). These grew optimally in sodium acetate buffer pH 6.0 with 2.4 *M* ammonium sulfate and 20% glycerol, regularly diffracting to ∼1.3 Å resolution in space group *C*222_1_. Type 2 crystals displayed high anisotropy, but were otherwise free from crystal pathology. Under lower pH, higher glycerol and lower ammonium sulfate conditions, type 2 crystals tended not to form, allowing the formation of type 3 crystals (Supplementary Fig. S1*c*), which grew as rectangular prisms of as large as 500 × 500 × 200 µm. Type 3 crystals consistently diffracted to ∼1.2 Å resolution in space group *P*4_3_2_1_2 with no crystal pathologies.

Initial attempts to solve the structure of *Mg*GH51 using data sets collected from type 1 and type 2 crystals by molecular replacement were unsuccessful. We attribute this failure to the poor sequence similarity between bacterial and fungal GH51 α-l-arabinofuranosidases. Alignment of *Mg*GH51 with its five closest characterized bacterial homologues (*E* < 10^−5^) using *MUSCLE* (Edgar, 2004 ▸) found 21–24% sequence identity (Supplementary Table S1). Attempts to solve the structure of *Mg*GH51 using *ab initio* methods were also unsuccessful. We attribute this failure to the relatively large size of the enzyme monomer. The failure to detect the substructure using bromine SAD was more surprising as all collected data sets had an overall CC_anom_ of >0.3. Subsequent analysis of the anomalous map derived from the bromine SAD data sets solved by molecular replacement with the sulfur SAD model clearly showed 14 partial occupancy structured bromide ions with anomalous map peak heights above 5σ, including three with peak heights above 10σ. However, this diffuse set of peaks was apparently insufficient to unambiguously determine phases.

Thus, the structure of *Mg*GH51 was solved by leveraging the unique capabilities of beamline I23 at DLS to measure anomalous signal at a wavelength of 2.75 Å *in vacuo*. Although the anomalous signal was weak (overall CC_anom_ = 0.13), it was robust across resolution bins and consistent in all data sets. Combining two 360° data sets gave an anomalous multiplicity of 15.2, an anomalous mean *I*/σ(*I*) of 0.82 and an overall mean *I*/σ(*I*) of 32.4. This weak anomalous signal proved sufficient for *SHELXD* to identify an unambiguous collection of peaks with a CFOM of 49.6 using a resolution cutoff of 2.78 Å. Subsequent refinement of these phases followed by chain-tracing using *ARP*/*wARP* correctly identified the position of every amino-acid residue in the known sequence (confirmed by N-terminal amino-acid sequencing; data not shown). Initially, owing to the failure to identify two *cis*-proline residues, three chains were traced, but these proline residues were manually remodelled and the chains were merged to complete the peptide-backbone structure. All other collected data sets were then readily solved by molecular replacement using this model.

### 3.2. The overall structure of *Mg*GH51   

**Figure 2:**
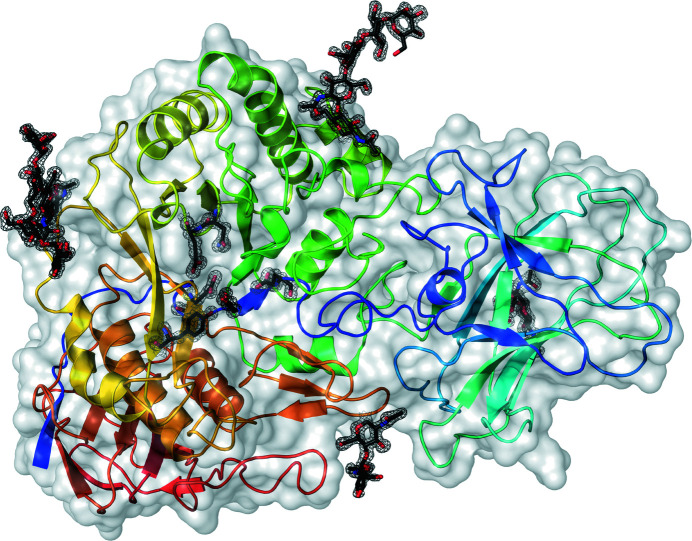
Overview of the tertiary structure of *Mg*GH51. The backbone of the polypeptide chain (PDB entry 6zpv) is shown in cartoon representation coloured from blue to red from the N-terminus to the C-terminus. The solvent-accessible surface of the enzyme is shown as a transparent grey surface. The observed high-mannose N-glycans, the side chains of key active-site residues (Glu427, Glu349, Asn348, Tyr400 and Glu21) and a glycerol molecule observed in the active-site pocket are shown as sticks with black C atoms. 2*F*_o_ − *F*_c_ electron density contoured at 1.5σ is shown around the sticks.

The overall fold of the catalytic domain of *Mg*GH51 is a (β/α)_8_ domain with a 12-stranded β-sandwich, typical of GH51 (Fig. 2 ▸). As in other GH51 enzymes, the N- and C-termini of the sequence are found in the 12-stranded β-sandwich domain, but unlike bacterial GH51 enzymes *Mg*GH51 has a 169-residue ten-stranded β-sandwich domain inserted following the 36-residue N-terminal strand. Density for four units of the N-glycan chitobiosyl ‘core’ was clearly visible, extending from Asn145, Asn245, Asn421 and Asn487 in all structures. Furthermore, for type 2 and type 3 crystals mannose residues could be clearly modelled extending from the cores of two glycans that were involved in crystal contacts.

Calculating the anomalous map for type 3 crystals revealed a collection of structured peaks on the surface of the protein. Comparable anomalous signal could be expected from either sulfate molecules or chloride ions. A variety of factors, including the distance between the anomalous peak and the protein backbone, near-coincidence with a well defined water molecule and the overall shape of the *F*_o_ − *F*_c_ map shape, led us to interpret 17 of these peaks (with anomalous peak heights of 5–10σ) as partial occupancy chloride ions. These chloride ions could not be reliably modelled into any other data set because of the lack of anomalous signal. Two acetate molecules were identified in type 3 crystals based on their shape, adjacency to cationic residues and lack of anomalous map density.

All three crystal forms arose from distinct collections of intermolecular interactions; however, both types 2 and 3 share a crystal-packing interface which obscures the active-site cleft (Supplementary Figs. S2*b* and S2*c*). This contrasts with type 1, which packs with a fully solvent-exposed active-site cleft (Supplementary Fig. S2*a*). Furthermore, a single molecule of glycerol was identified in the putative active-site pocket of *Mg*GH51 in type 2 and type 3 crystals, while only water was found in the active-site pocket of type 1 crystals, leading us to perform ligand-binding studies using type 1 crystals.

### 3.3. Active-site structure and conformational itinerary of *Mg*GH51   

**Figure 3:**
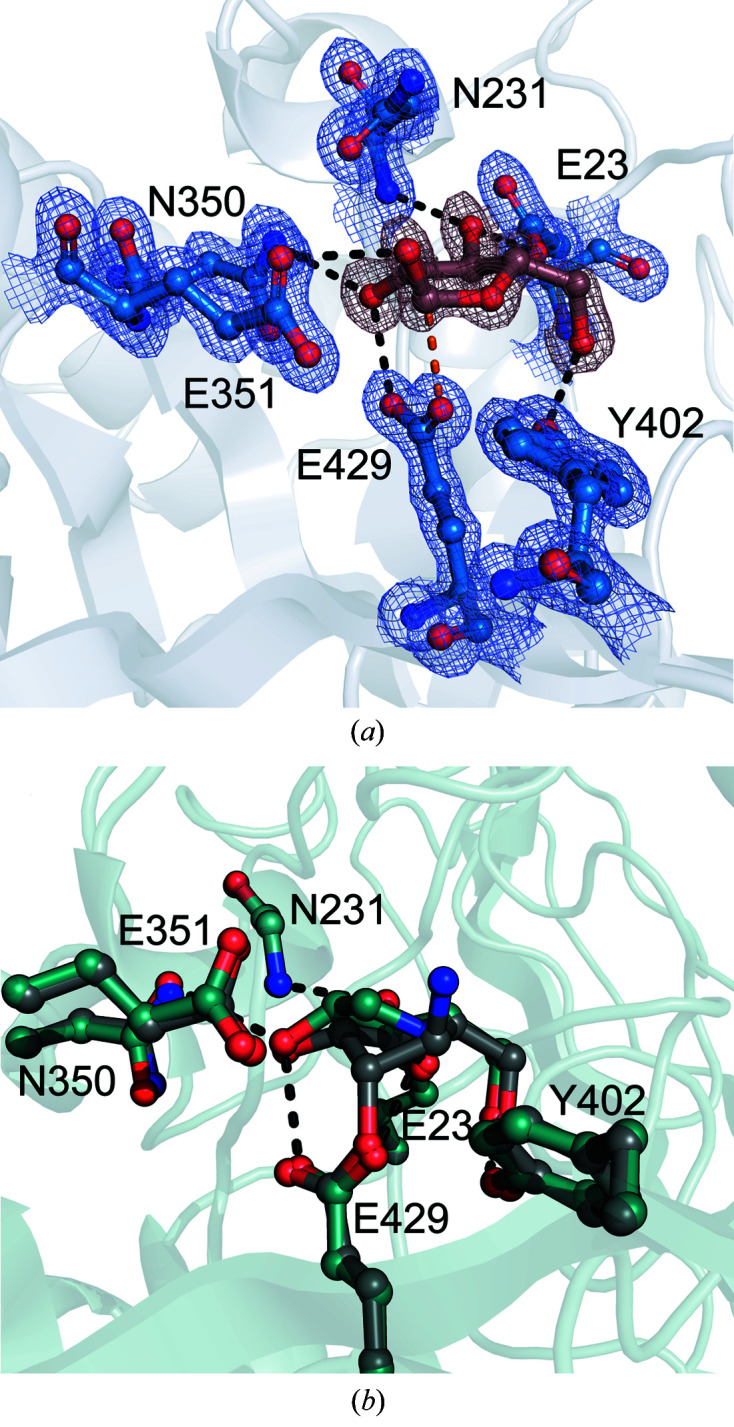
Active site and conformational itinerary of *Mg*GH51. (*a*) Active-site arrangement of *Mg*GH51 around α-l-arabinofuranose. Active-site residues interacting with α-l-arabinofuranose (brown) are shown as blue sticks. Apparent hydrogen-bonding interactions are shown as black dashed lines. The interaction between the catalytic nucleophile and the anomeric C atom is shown as an orange dashed line. Electron density is shown as a mesh contoured at 1.5σ. (*b*) Overlay of the AraDNJ and α-l-AraAZI ligands within the *Mg*GH51 active site. The active-site residues and ligand from the α-l-AraAZI complex are shown as grey sticks. The active-site residues and ligand from the AraDNJ complex are shown as cyan sticks. Apparent hydrogen-bonding interactions are shown as black dashes.

A 1.27 Å resolution data set collected from a type 1 *Mg*GH51 crystal soaked with α-l-arabinose was solved with clear density for a single molecule of α-l-arabinose in the active-site pocket (Fig. 3 ▸ *a*). The positioning of the monosaccharide within the active site revealed key enzyme–product interactions, including the hydrogen-bonding interactions Tyr402–O5, Glu23–O3, Asn231–O3, Asn350–O2, Glu429–O2 and Glu351–O1. Glu429 was positioned directly below the anomeric C atom, suggesting that it is the catalytic nucleophile, while the Glu351–O1 interaction suggested that it is the general acid/base. The bound arabinose is found with a ^4^ *T*_3_ ring conformation, matching the reported conformation of α-l-arabinose bound in the active site of *T. maritima* GH51 (PDB entry 3ug4; Im *et al.*, 2012 ▸).

AraDNJ is a well known reversible α-l-arabinofurano­sidase inhibitor. Often thought to mimic the oxocarbenium-ion transition state of α-l-arabinofuranoside cleavage (Gloster *et al.*, 2007 ▸), this inhibitor has recently been applied in the understanding of the mechanism of GH62 α-l-arabinofuranosidases (Moroz *et al.*, 2018 ▸), where it bound in a Michaelis complex-like ^4^ *E* conformation. Solving the structure of *Mg*GH51 in the presence of AraDNJ gave a 1.70 Å resoljution structure with clear density for AraDNJ in the active site. Retaining the interactions observed between l-arabinose and Tyr402, Glu23, Asn231 and Asn350, this structure revealed an apparent binding of Glu429 across O2 and the ring N atom, causing the ring to pucker into a ^4^ *E* ring conformation like that observed in GH62, not the *E*_3_ conformation expected for the transition state.

Soaking *Mg*GH51 crystals with stoichiometric α-l-AraCS or α-l-AraAZI gave clear density for these two ligands bound to Glu429, confirming its role as catalytic nucleophile (Fig. 3 ▸ *b*). Clear density was present for the primary amine or sulfate group on C6 resulting from the ring-opening addition reaction (Supplementary Fig. S3). While the steric bulk of the sulfate group of α-l-AraCS caused some rearrangement of the active site, the addition of α-l-AraAZI to Glu429, leaving a primary amine in place of the sulfate, caused no apparent changes to the protein structure relative to the unliganded enzyme, suggesting that this structure is a good mimic for the natural glycosyl-enzyme intermediate. In line with observations of the complex between the same inhibitor and *Gs*GH51, a bacterial GH51 (McGregor *et al.*, 2020 ▸), the furanose ring conformation of the complex is ^2^ *E*. *Mg*GH51 follows a mechanism that runs through the same conformational itinerary used by bacterial GH51 α-l-arabinofuranosidases. Thus, the conformational itinerary of GH51 α-l-arabinofuranosidases appears to be conserved across kingdoms of life.

Interactions with the larger arabinoxylan substrate are of particular interest because of the inability of *Mg*GH51 to hydrolyse l-arabinose residues from doubly substituted d-xylose residues (Sørensen *et al.*, 2006 ▸). For this enzyme, the α-l-arabinofuranose bound into the enzyme active site with O1 pointing directly out of the active site into a narrow cleft. We hypothesized that this cleft would bind xylooligosaccharides. However, a soak with xylopentaose gave a type 1 crystal structure with no apparent additional density in this active-site-adjacent cleft (data not shown). Thus, the exact nature of positive subsite interactions in fungal GH51 α-l-arabinofuranosidases remains obscure, although active-site homology provides a basis on which the nature of the interactions between the enzyme and the xylan backbone may be inferred (see below).

### 3.4. Homology to bacterial GH51 α-l-arabinofuranosidases   

**Figure 4:**
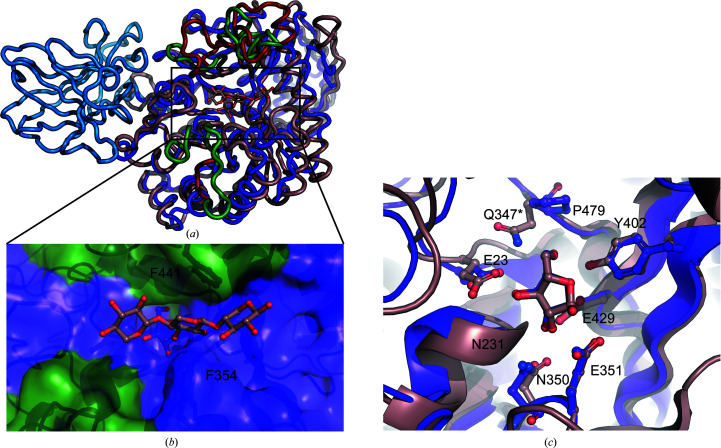
Homology of *Mg*GH51 to *Tx*GH51. (*a*) Loop representation of the structure of *Mg*GH51 superimposed with *Tx*GH51. For *Mg*GH51, the catalytic domain is shown in purple, the domain insertion in *Mg*GH51 is shown in blue and the remodelled loops are shown in green. For *Tx*GH51, the catalytic domain is shown in tan and the remodelled loops (or sites of loop insertion) are shown in red. The ligand bound to *Tx*GH51 is shown in ball-and-stick representation. (*b*) Close-up view of the substrate-binding cleft of *Mg*GH51 in surface representation with the same colouring as in (*a*). The l-­arabinofuranose observed bound to *Mg*GH51 is shown in purple and the three xylose residues observed in the *Tx*GH51 structure are superposed onto the structure of *Mg*GH51 to show the expected binding position of the xylan backbone. Phe354 and Phe441 are labelled and shown as sticks below the transparent surface. (*c*) Close-up view of the superimposed active sites of *Tx*GH51 and *Mg*GH51. Both enzymes are shown as cartoon representations with active-site residues and the bound l-arabinofuranose shown as ball-and-stick representations with the same colouring as in (*a*). Residue names are from *Mg*GH51 unless marked with a star. The *Tx*GH51 residue homologous to Glu351 was engineered to be Gln in place of the wild-type Glu.

The most significant difference between *Mg*GH51 and its bacterial homologues is the presence of the additional N-terminal β-sandwich domain (Fig. 4 ▸ *a*, blue). A *DALI* (Holm & Laakso, 2016 ▸) search using this domain in isolation found some structural similarity (r.m.s.d. of 2.7–2.8 Å, 14% sequence identity) to CBM4 carbohydrate-binding modules, notably PDB entries 2y6g and 3k4z, both of which are cellulose-binding domains (von Schantz *et al.*, 2012 ▸; Alahuhta *et al.*, 2010 ▸). Such domains are common components of biomass-degrading enzymes (Boraston *et al.*, 2004 ▸). However, this CBM4-like domain is atypical, containing no apparent binding cleft or aromatic binding platform. To look for signs of a cellulose-binding site, crystals were soaked with 10 m*M* cellohexaose, yielding a type 1 structure with no apparent additional density (data not shown). Thus, it does not appear that this domain has a xylan- or cellulose-binding site and it does not appear to be an arabinofuranose-binding domain akin to that found in *A. kawachii* GH54 (Miyanaga *et al.*, 2004 ▸). The purpose of this inserted domain remains unclear.

A simple sequence alignment between *Mg*GH51 and *T. xylanilyticus* GH51 (*Tx*GH51; PDB entry 2vrq; Paës *et al.*, 2008 ▸), a structurally characterized bacterial homologue (*DALI* *Z*-score of 35.2, r.m.s.d. of 2.5 Å), finds only 23% sequence identity (Supplementary Table S1). However, structural superposition of *Mg*GH51 onto *Tx*GH51 using only arabinofuranose, the general acid/base and the catalytic nucleophile reveals significant overall structural homology (Fig. 4 ▸ *b*). The active sites of the two enzymes are nearly identical, with absolute conservation of Tyr402 (Tyr242 in *Tx*GH51), Glu23 (Glu28 in *Tx*GH51), Asn350 (Asn175 in *Tx*GH51), the Asn231 backbone (the Cys74 backbone in *Tx*GH51), Glu351 (Glu176 in *Tx*GH51) and Glu429 (Glu298 in *Tx*GH51). The only polar contact that is not conserved between the two active sites is the interaction between Gln347 and O5 found only in *Tx*GH51.

Structural alignment also reveals several areas of significant restructuring around the active site. Below the active-site cleft *Mg*GH51 has two loop insertions, and above the active-site cleft *Mg*GH51 has two extended and significantly remodelled loops (Fig. 4 ▸ *a*, green). Together, these differences result in a significantly more restricted active-site cleft. Superimposition of the three xylose residues observed in PDB entry 2vrq onto the model of *Mg*GH51 suggests that the observed restructuring does not interfere with xylan-chain binding but may restrict access to the active site for bulkier, more highly branched polysaccharides (Fig. 4 ▸ *c*). Indeed, it has been reported that *Mg*GH51 is unable to cleave α-l-arabino­furanose residues from doubly substituted xylose residues (Sørensen *et al.*, 2006 ▸). The superimposition of xylose residues from the structure of *Tx*GH51 reveals that, with α-l-arabinofuranose extending from O3, there is no pocket which could accommodate an α-l-arabinofuranose residue extending from O2 and that O2 is likely to form a hydrogen bond with Asp351 when unsubstituted (Fig. 4 ▸ *a*). This superimposition also suggests that interactions with the polysaccharide backbone in the +1 subsite occur primarily through hydrophobic stacking interactions with Phe354 and Phe441, a structural motif analogous to that which is thought to be responsible for the broad substrate specificity of *Ct*Araf51A (Taylor *et al.*, 2006 ▸). Measurement of the hydrolytic kinetics of *Mg*GH51 towards the AX2 and A3 oligosaccharides confirmed the presence of only a fourfold specificity for xylose over arabinose in the positive subsites (Supplementary Fig. S4 and Table S1).

Owing to the relatively high degree of sequence similarity among fungal GH51 enzymes (Supplementary Table S3), reliable sequence alignments can be constructed for other functionally characterized fungal GH51 enzymes (Supplementary Fig. S5). In addition, the structure of *Mg*GH51 presented here facilitates the construction of fungal GH51 homology models. To investigate a possible molecular rationale for the observation that some fungal GH51 enzymes, notably *A. niger* GH51 (*An*GH51), have detectable activity towards l-arabinose on double-substituted xylose residues (Koutaniemi & Tenkanen, 2016 ▸), we constructed a structural model of *An*GH51 using *SWISS-MODEL* with the *Mg*GH51 template (Waterhouse *et al.*, 2018 ▸; Supplementary Fig. S6). Comparison of the modelled active-site cleft of *An*GH51 with the active-site cleft of *Mg*GH51 shows a high degree of conservation. However, Phe353 and Phe354 of *Mg*GH51 are replaced by methionine and leucine, while a loop below the active site has been deleted. This has the cumulative effect of significantly opening up the active site adjacent to O2 of the +1 xylose residue, possibly allowing a strained, but productive, inter­action. We believe that this combination of mutations and loop deletion is likely to be responsible for the observed trace activity. Indeed, this FF→ML mutation is conserved among other functionally characterized fungal GH51 enzymes, while the deletion of the 433–442 loop is unique to GH51 enzymes from *Aspergillus*, which are the only known fungal GH51 enzyme with this trace activity (Koutaniemi & Tenkanen, 2016 ▸). A broader examination of sequence conservation among 100 fungal GH51 enzymes using *ConSurf* (Ashkenazy *et al.*, 2016 ▸) shows that the amino-acid residues forming the active site and core fold of the protein are highly conserved, but that there is significantly higher variability in the loops above and below the active-site cleft (Supplementary Fig. S7), as well as in the inserted N-terminal domain, suggesting that while the core function of this family is conserved, there is significant diversity in how it recognizes and tolerates polysaccharide substrates.

In summary, we have presented the structure of *Mg*GH51, a fungal GH51 α-l-arabinofuranosidase, which was solved using sulfur SAD data collected on the long-wavelength *in vacuo* beamline I23 at Diamond Light Source. We have shown that despite poor overall sequence conservation and the insertion of an additional domain of as yet unknown function, *Mg*GH51 shares a conserved active-site architecture with bacterial homologues. We show that the active-site cleft of *Mg*GH51 shares other key features with bacterial homologues, such as the hydrophobic clamp in the +1 subsite, and that it lacks a pocket which could facilitate the recognition of disubstituted substrates, explaining its observed specificity. Through sequence analysis and homology modelling, we have shown that fungal GH51 enzymes display significant sequence diversity concentrated in the loops above and below the active-site cleft, offering a molecular basis for the observed differences in substrate specificity reported for these essential biomass-degrading enzymes.

## 4. Related literature   

The following reference is cited in the supporting information for this article: Robert & Gouet (2014 ▸).

## Supplementary Material

PDB reference: *Mg*GH51, unliganded, crystal type 1, 6zpy

PDB reference: crystal type 2, 6zpx

PDB reference: crystal type 3, 6zpv

PDB reference: crystal type 3, collected at 2.75 Å wavelength, 6zps

PDB reference: complex with α-l-AraCS, crystal type 1, 6zpw

PDB reference: complex with arabinose, crystal type 1, 6zpz

PDB reference: complex with α-l-AraAZI, crystal type 1, 6zq0

PDB reference: complex with AraDNJ, crystal type 1, 6zq1

Supplementary Tables and Figures. DOI: 10.1107/S205979832001253X/dw5211sup1.pdf

## Figures and Tables

**Table 1:** Macromolecule-production information

Source organism	*Meripilus giganteus*
Expression host	*Aspergillus oryzae*
Complete amino-acid sequence of the construct produced	VTVTVNKNPSHTVPSTLYGLMFEDINHSGDGGLYAELLQNRAFQQVTPNTAAALAAWHPISNAKLAVIQDPSPVSNALPNSLQFSVPSGSSGRVGFTNEGFWGIKVDSTWTYKASLFFRFPTSSSFSGALTVGLQTNAGRVLAQNSTQIRGTTTKWTQINLELHPTASAPDVSNSFFVTIDGAAGAGQTINFAMFSLFPPTFKNRPNGLRADIAETLAEMGPSFFRFPGGNNLEGQTTATRWQWNATVGSLLDRPGRVGDWGYVNTDGLGLLEYLQFFEDTGMEPIMAVWAGYSLGGTSLAENQLAPYIQQAIDQINFVIGDPAKSAPAALRASLGHPEPFTLRFVEVGNEDFFAAGSYPYRWHDFVTALQAQFPQIRFIATTNAWNPVLSPVPQSYDVHVYQTPTWFYQNAFYYDGFQRNGTTYFEGEYAAISTNANDLFGTVADGRLAFPTVQSATGEAAFMTGLERNSDIVFAASYAPLLQHVNSTQWTPDLVSYDAGSVIKSTSFFAQKLFALNKGDQYLPSTLPTNGGTLHWSITRASSSGKTFIKIANAGSSAQSLTFQLTQFNSVSSTGTLQVLTGPETASNTPEAPQAIVPKTSTIGTGKTFTYNAPAFSVSVITVTTN

**Table 2:** Crystallization

Method	Sitting drop	Sitting drop
Plate type	Swissci 48-well MRC Maxi	Swissci 48-well MRC Maxi
Temperature (K)	293	293
Protein concentration (mg ml^−1^)	10	10
Buffer composition of protein solution	10 m*M* sodium acetate pH 5.5, 100 m*M* NaCl	10 m*M* sodium acetate pH 5.5, 100 m*M* NaCl
Composition of reservoir solution	20% PEG 3350, 0.1 *M* bis-Tris–HCl pH 6.5, 0.2 *M* NaNO_3_	1.8–2.4 *M* (NH_4_)_2_SO_4_, 0.1 *M* sodium acetate pH 5–6, 20–35% glycerol
Volume and ratio of drop	1.8 µl, 2:1 PS:RS	1.8 µl, 2:1 PS:RS
Volume of reservoir (µl)	100	100

**Table 3:** Data collection and processing Values in parentheses are for the outer shell.

	Crystal type 3	Crystal type 3	Crystal type 2	Crystal type 1	L-Arabinose complex	α-L-AraCS complex	α-L-AraAZI complex	AraDNJ complex
PDB code	6zpv	6zps	6zpw	6zpx	6zpy	6zpz	6zq0	6zq1
Diffraction source	I23, DLS	I23, DLS	I03, DLS	I03, DLS	I04-1, DLS	I04-1, DLS	I04-1, DLS	I04, DLS
Wavelength (Å)	1.3776	2.7552	0.9763	0.9763	0.9119	0.9119	0.9119	0.9795
Temperature (K)	80	80	100	100	100	100	100	100
Detector	PILATUS 2M	PILATUS 2M	EIGER2 XE 16M	EIGER2 XE 16M	PILATUS 6M-F	PILATUS 6M-F	PILATUS 6M-F	EIGER2 XE 16M
Crystal-to-detector distance (mm)	10†	10†	175	175	214	214	214	278
Rotation range per image (°)	0.1	0.1	0.1	0.1	0.1	0.1	0.1	0.1
Total rotation range (°)	360	360	220	220	220	220	220	360
Exposure time per image (s)	0.1	0.1	0.01	0.01	0.04	0.04	0.04	0.01
Space group	*P*4_3_2_1_2	*P*4_3_2_1_2	*C*222_1_	*P*2_1_2_1_2_1_	*P*2_1_2_1_2_1_	*P*2_1_2_1_2_1_	*P*2_1_2_1_2_1_	*P*2_1_2_1_2_1_
*a*, *b*, *c* (Å)	83.95, 83.95, 256.59	84.17, 84.17, 257.35	114.95, 125.84, 161.23	59.98, 65.80, 193.12	59.71, 65.04, 174.12	58.03, 65.61, 191.45	59.050, 65.460, 191.820	59.69, 66.04, 193.83
α, β, γ (°)	90, 90, 90	90, 90, 90	90, 90, 90	90, 90, 90	90, 90, 90	90, 90, 90	90, 90, 90	90, 90, 90
Mosaicity (°)	0.10	0.10	0.16	0.40	0.31	0.27	0.14	0.23
Resolution range (Å)	85.53–1.20 (1.22–1.20)	257.35–1.79 (1.83–1.79)	80.61–1.33 (1.35–1.33)	82.28–1.30 (1.32–1.30)	65.04–1.27 (1.29–1.27)	58.12–1.71 (1.74–1.71)	61.95–1.54 (1.57–1.54)	29.87–1.70 (1.73–1.70)
Total No. of reflections	9407615 (350182)	5201021 (99589)	2132590 (87779)	1461368 (55378)	1394136 (62439)	602802 (25957)	849481 (34963)	2941195 (71749)
No. of unique reflections	285059 (13929)	74070 (4624)	265496 (12835)	188207 (9226)	176357 (8584)	79871 (3871)	110936 (5472)	85172 (4409)
Completeness (%)	100 (100)	84.4 (90.5)‡	99.9 (98.0)	100 (99.7)	98.5 (97.3)	98.8 (98.5)	99.7 (99.7)	99.9 (99.4)
Multiplicity	33 (25.1)	70.2 (21.5)	8.0 (6.8)	7.8 (6.0)	7.9 (7.3)	7.5 (6.7)	7.7 (6.4)	34.5 (16.3)
〈*I*/σ(*I*)〉	27.2 (1.8) [1.24 Å]§	35.1 (5.7)	9.6 (1.1) [1.42 Å]§	6.9 (1.2) [1.38 Å]§	7.4 (0.8) [1.41 Å]§	8.7 (1.2) [1.90 Å]§	10.6 (0.9) [1.75 Å]§	15.9 (2.1)
CC_1/2_	1.000 (0.661)	0.999 (0.859)	0.999 (0.521)	0.998 (0.778)	0.996 (0.764)	0.997 (0.419)	0.999 (0.505)	0.999 (0.915)
*R*_r.i.m._	0.011 (0.430)	0.012 (0.142)	0.035 (0.727)	0.035 (0.422)	0.040 (0.374)	0.052 (0.694)	0.043 (0.731)	0.028 (0.281)
Overall *B* factor from Wilson plot (Å^2^)	12.23	16.98	11.86	13.16	11.14	21.04	18.22	13.52

† Distance from the central axis of the cylindrical detector array.

‡ Completeness is low owing to the cylindrical shape of the detector and the long wavelength that was used.

§ Data in the outer shell were cut using a CC_1/2_ limit of 0.5 or *I*/σ(*I*) > 1. The resolution at which *I*/σ(*I*) falls below 2.0 is provided in square brackets.

**Table 4:** Structure solution and refinement Values in parentheses are for the outer shell.

	Crystal type 3	Crystal type 3	Crystal type 2	Crystal type 1	L-Arabinose complex	α-L-AraCS complex	α-L-AraAZI complex	AraDNJ complex
PDB code	6zpv	6zps	6zpw	6zpx	6zpy	6zpz	6zq0	6zq1
Resolution range (Å)	79.918–1.200 (1.231–1.200)	80.128–1.795 (1.841–1.795)	80.743–1.329 (1.364–1.329)	62.362–1.300 (1.334–1.300)	56.54–1.27 (1.30–1.27)	55.65–1.71 (1.75–1.71)	62.03–1.54 (1.58–1.54)	62.47–1.70 (1.74–1.70)
Completeness (%)	100.0 (100.0)	84.5 (92.1)	99.9 (98.5)	99.9 (99.6)	98.4 (96.8)	99.4 (98.8)	99.9 (99.8)	99.9 (99.3)
No. of reflections, working set	270778 (19735)	70385 (5592)	252355 (18312)	178687 (13150)	167555 (12126)	75804 (5523)	105323 (7706)	80757 (5920)
No. of reflections, test set	14149 (1063)	3570 (270)	13118 (937)	9384 (629)	8652 (593)	3991 (276)	5515 (385)	4303 (264)
Final *R*_cryst_	0.121 (0.227)	0.145 (0.160)	0.130 (0.266)	0.180 (0.340)	0.141 (0.293)	0.189 (0.304)	0.180 (0.321)	0.196 (0.297)
Final *R*_free_	0.144 (0.247)	0.178 (0.212)	0.151 (0.276)	0.217 (0.333)	0.179 (0.306)	0.225 (0.297)	0.206 (0.314)	0.216 (0.313)
Cruickshank DPI (Å)	0.024	0.090	0.030	0.049	0.041	0.101	0.070	0.100
No. of non-H atoms								
Total	6281	5974	5807	5598	5827	5541	5608	5471
Protein	4915	4917	4857	4832	4849	4835	4824	4793
Ligand	269	278	221	123	153	137	133	132
Water	1097	762	725	643	824	568	650	545
Ion	0	17	1	1	1	1	1	1
R.m.s. deviations								
Bond lengths (Å)	0.018	0.013	0.014	0.016	0.015	0.012	0.012	0.11
Angles (°)	2.1	1.8	1.9	1.9	1.8	1.7	1.8	1.7
Average *B* factors (Å^2^)								
Protein	14.7	19.7	15.4	19.7	15.2	25.0	20.7	20.6
Ligand	30.2	34.3	28.7	31.3	25.8	37.6	31.7	32.0
Water	29.7	31.1	28.3	29.1	26.3	31.5	29.3	27.2
Ion	—	41.7	15.4	33.4	18.24	31.4	31.3	26.1
Ramachandran plot								
Most favoured (%)	97.9	98.4	98.1	97.9	98.4	97.3	98.2	97.6
Allowed (%)	100	100	100	100	100	100	100	100
