# Supplementary material for: Structure of a GH51 α-l-arabinofuranosidase from Meripilus giganteus: conserved substrate recognition from bacteria to fungi
Source: Acta Crystallogr D Struct Biol. 2020 Oct 16;76(Pt 11):1124–33. doi: 10.1107/S205979832001253X (PMC7604909; doi:10.1107/S205979832001253X)
Supplement: Supplementary file 1 [file d-76-01124-sup1.pdf]

Acta Cryst D STRUCTURAL BIOLOGY

Volume 76 (2020)

Supporting information for article:

Structure of a GH51 α-L-arabinofuranosidase from *Meripilus giganteus*: conserved substrate recognition from bacteria to fungi

Nicholas G. S. McGregor, Johan P. Turkenburg, Kristian B. R. Mørkeberg Krogh, Jens Erik Nielsen, Marta Artola, Keith A. Stubbs, Herman S. Overkleeft and Gideon J. Davies

Table S1 Percent identity matrix for the multiple sequence alignment of *Mg*GH51 with its five closest structurally characterised homologues.

The PDB ID of each sequence is given in brackets next to the sequence name.

	*Mg*GH51	*Tx*GH51 (2VRQ)	*Tm*GH51 (3UG3)	*Tp*GH51 (3S2C)	*Gs*GH51 (1PZ3)	*Bl*GH51 (2Y2W)
*Mg*GH51 (6ZPV)	**100**	**22.8**	**24.2**	**24.3**	**21.0**	**23.6**
*Tx*GH51 (2VRQ)	22.8	100	33.9	33.5	26.8	28.7
*Tm*GH51 (3UG3)	24.2	33.9	100	99.4	35.8	33.8
*Tp*GH51 (3S2C)	24.3	33.6	99.4	100	35.7	33.5
*Gs*GH51 (1PZ3)	21.0	26.8	35.8	35.7	100	46.6
*Bl*GH51 (2Y2W)	23.6	28.7	33.8	33.5	46.6	100

Table S2 Kinetic parameters for the hydrolysis of oligosaccharides by *Mg*GH51

Substrate	k_{cat} (s^{-1})	K_M (mM)	K_{cat}/K_M ($M^{-1}s^{-1}$)
AX2	3900±200	1.4±0.1	$2.8 \cdot 10^6$
A3	4500±600	6.4±1.2	$7.0 \cdot 10^5$
A5	2000±100	3.7±0.3	$5.3 \cdot 10^5$

Table S3 Percent identity matrix for the multiple sequence alignment of *Mg*GH51 with six functionally characterised fungal homologues.

The GenBank accession code of the sequence is given for each sequence.

	*Mg*GH5 1	*An*GH5 1	*Ak*GH5 1	*Aa*GH5 1	*Pa*GH5 1	*Tpu*GH5 1	*Pc*GH5 1
*Mg*GH51_CAL81200.1	**100**	**40.6**	**41.3**	**41.3**	**35.7**	**36.5**	**36.6**
*An*GH51_CAK43424.1	40.6	100	97.3	97.5	39.2	38.8	40.3
*Ak*GH51_BAB96815.1	41.3	97.3	100	99.7	39.2	39.5	41.0
*Aa*GH51_BAB21568.2	41.3	97.5	99.7	100	39.1	39.5	41.0
*Pa*GH51_CAP62201.1	35.7	39.2	39.2	39.1	100	53.3	54.1
*Tpu*GH51_ABO93602. 1	36.5	38.8	39.5	39.5	53.3	100	78.2
*Pc*GH51_BAG71680.1	36.6	40.3	41.0	41.0	54.1	78.2	100

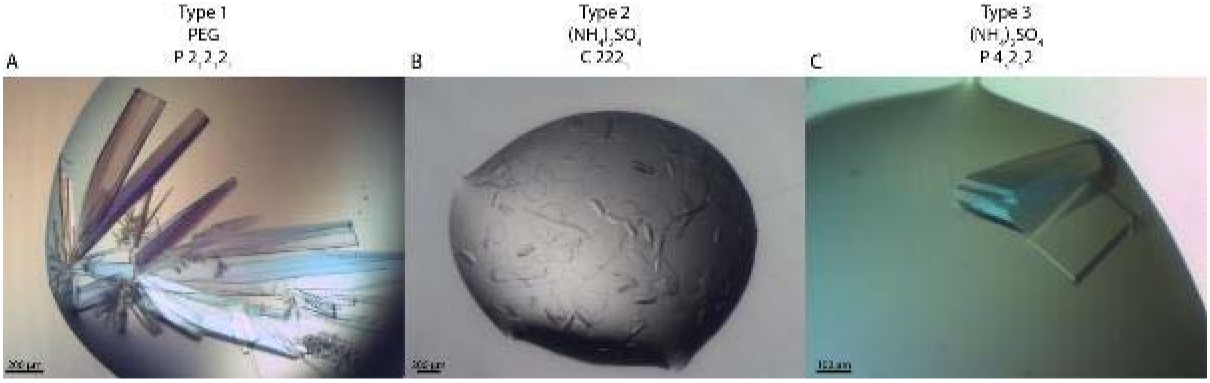

Figure S1 Images of *Mg*GH51 crystals. A) Image of type 1 crystals taken under polarized light. B) Image of type 2 crystals taken under non-polarized light. C) Image of type 3 crystals taken under polarized light.

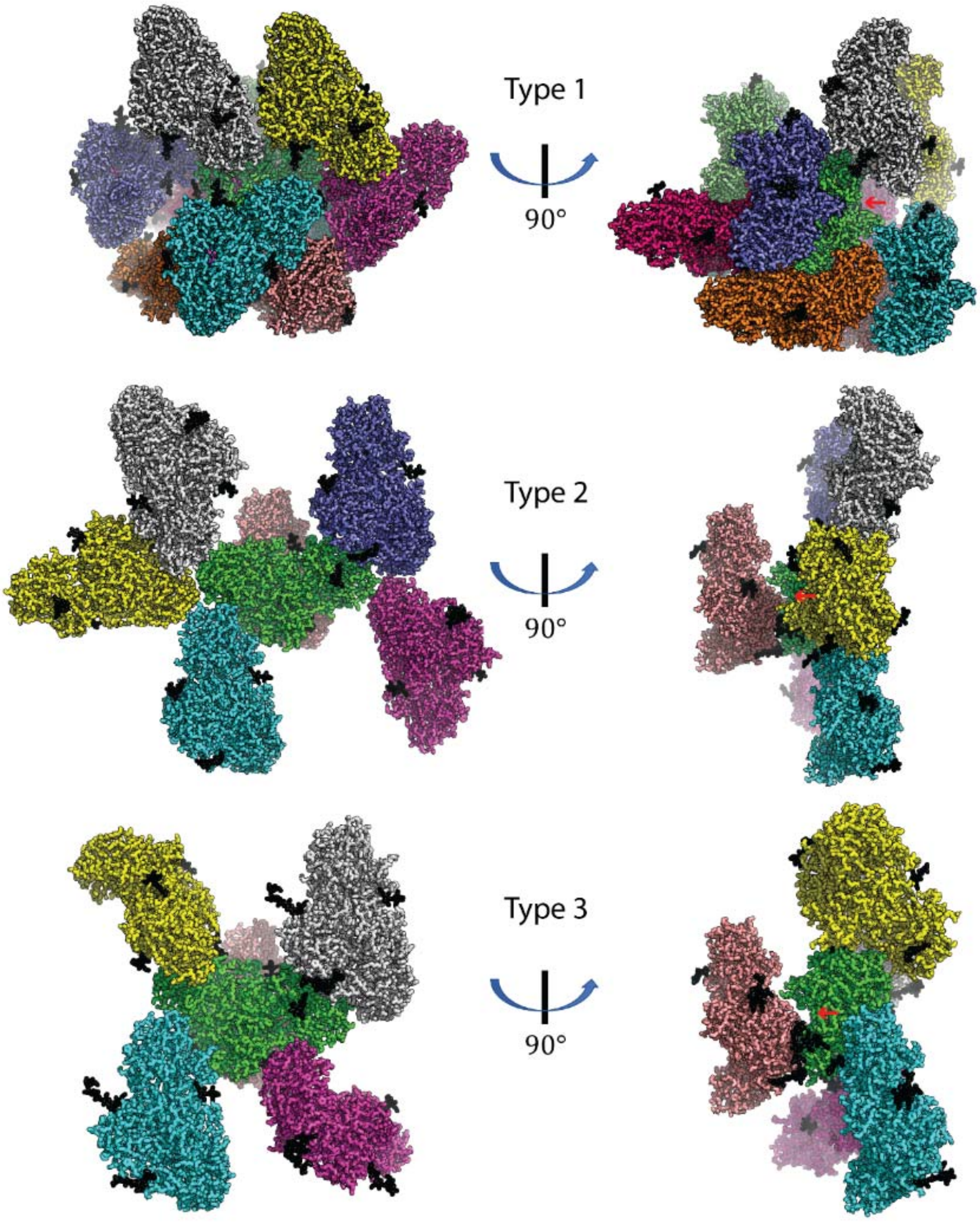

Figure S2 Crystal packing of *Mg*GH51. Symmetry-related molecules of *Mg*GH51 which form crystal contacts with a central copy of *Mg*GH51 (green) are shown for each crystal type. A red arrow marks the location of the active site of the green molecule. Glycans are shown in black.

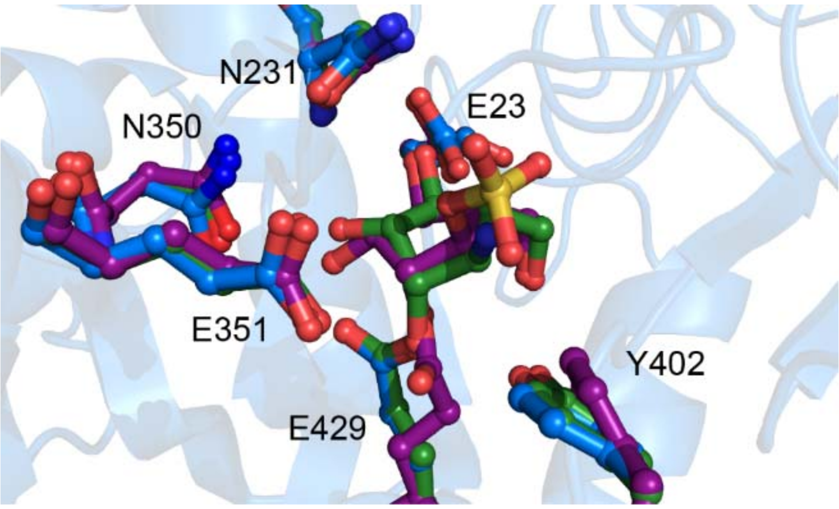

Figure S3 Superimposition of the α-L-AraAZI (green) and α-L-AraCS (purple) complex structures of *Mg*GH51 onto the unliganded structure (blue) showing the different positions of the sulphate and primary amine groups on C6, as well as the different positions of E429 and Y402 between the α-L-AraCS complex and the other complexes.

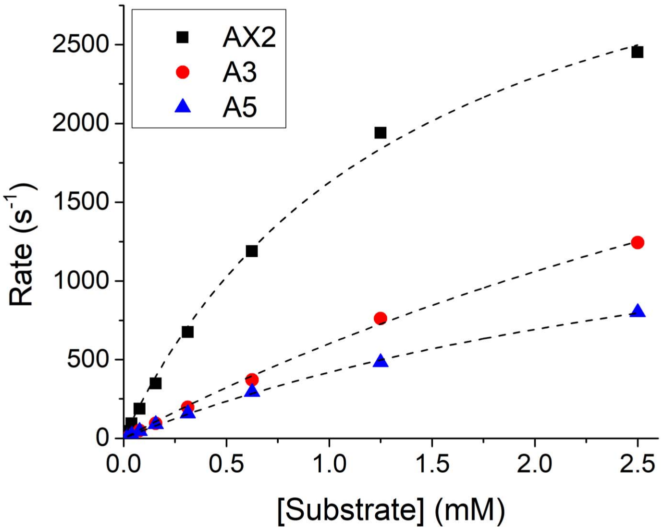

Figure S4 Arabinose release rate vs. substrate concentration for *Mg*GH51 acting on different α-L-arabinofuranose-containing oligosaccharides. Each point represents a single rate measurement.

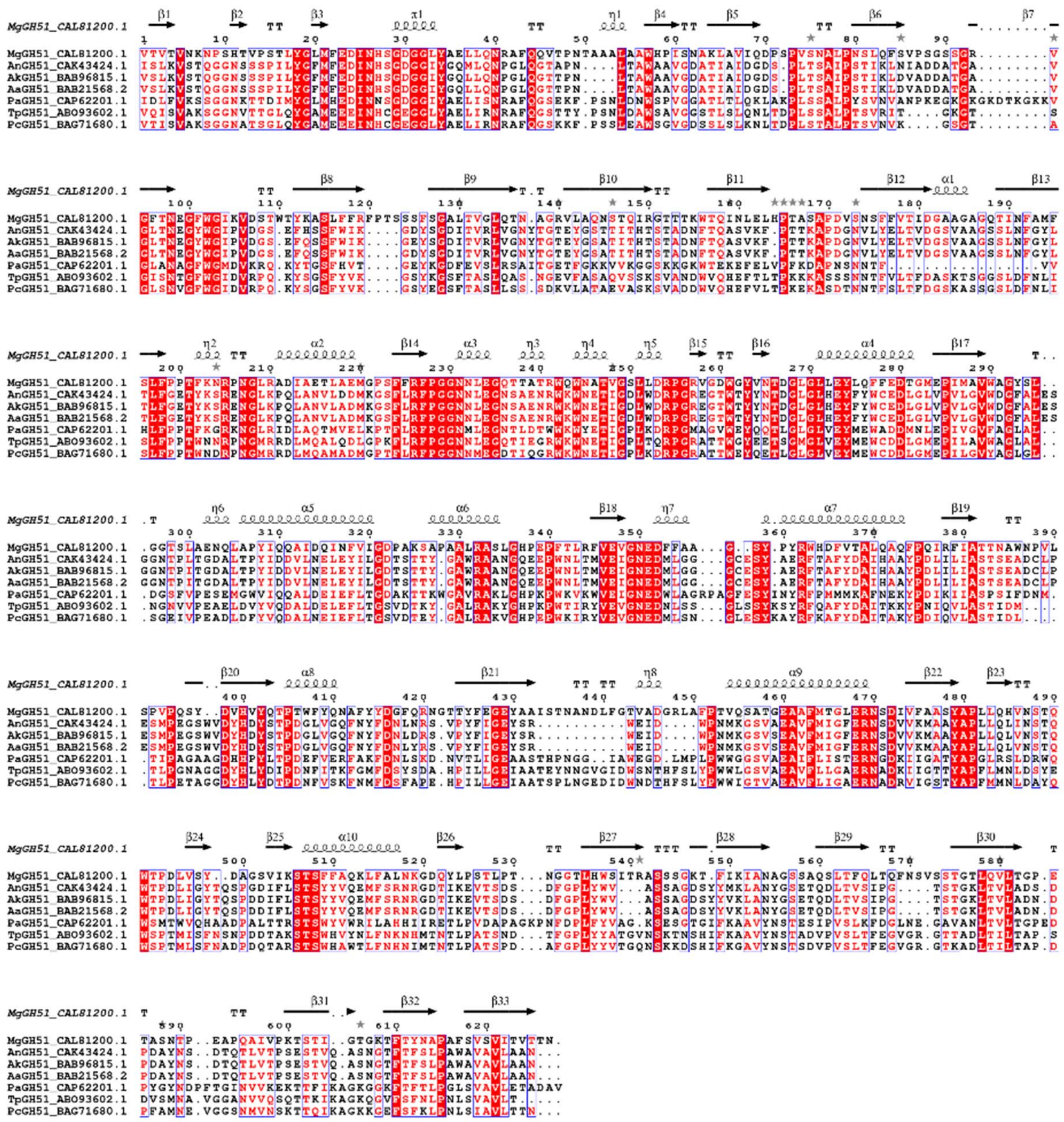

Figure S5 Amino acid sequence alignment of *Mg*GH51 with functionally characterised fungal GH51 enzymes. The GenBank accession code for each sequence is given beside its name. The secondary structure of *Mg*GH51 is shown above the sequence alignment. The alignment is displayed using ESPript (http://espript.ibcp.fr (Robert & Gouet, 2014)) with default settings and "thermal" display.

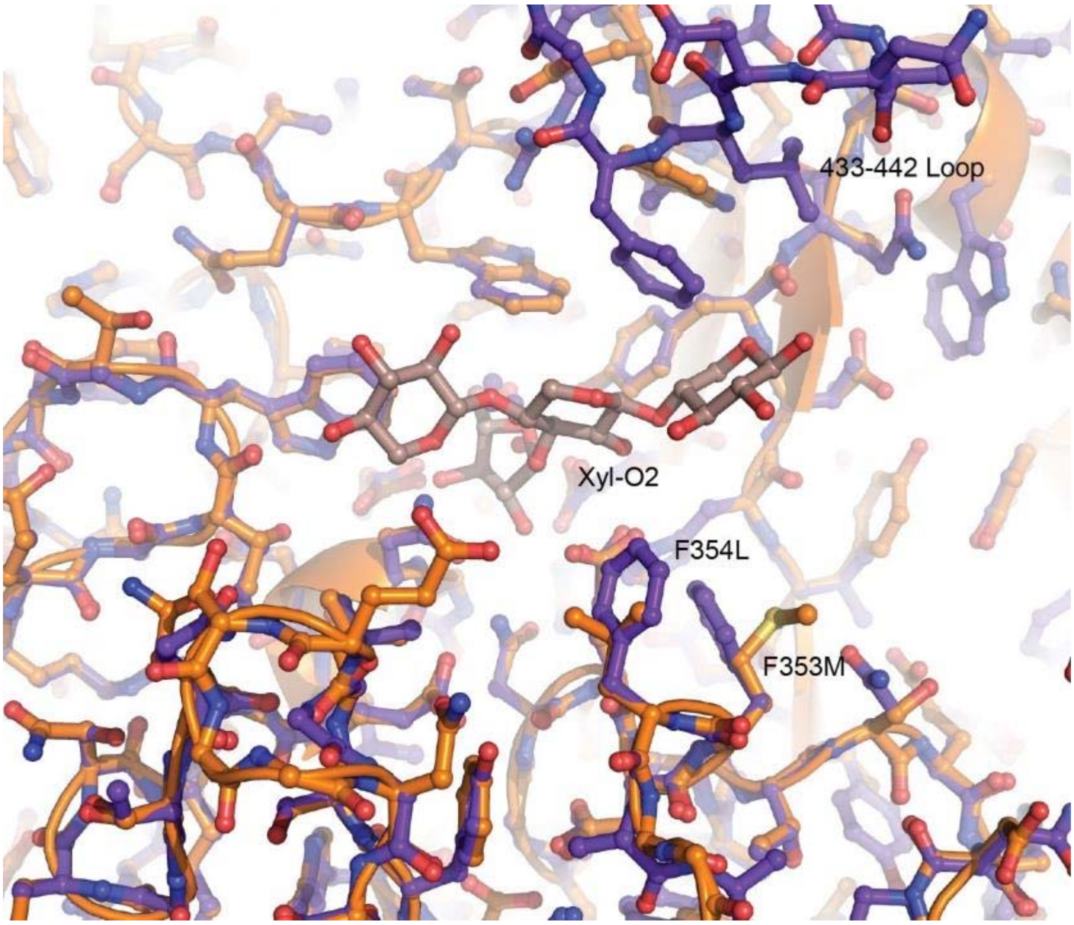

Figure S6 Superimposition of the homology model of *An*GH51 onto the structure of *Mg*GH51. The structure of *Mg*GH51 is shown in purple, the structural model of AnGH51 is shown in orange and the xylose residues from *Tx*GH51 are shown as in Figure 4. The positions of key differences between *Mg*GH51 and *An*GH51, including the F353M and F354L mutations, and the deleted 433-442 loop, are noted in relation to the position of O2 of the +1 xylose.

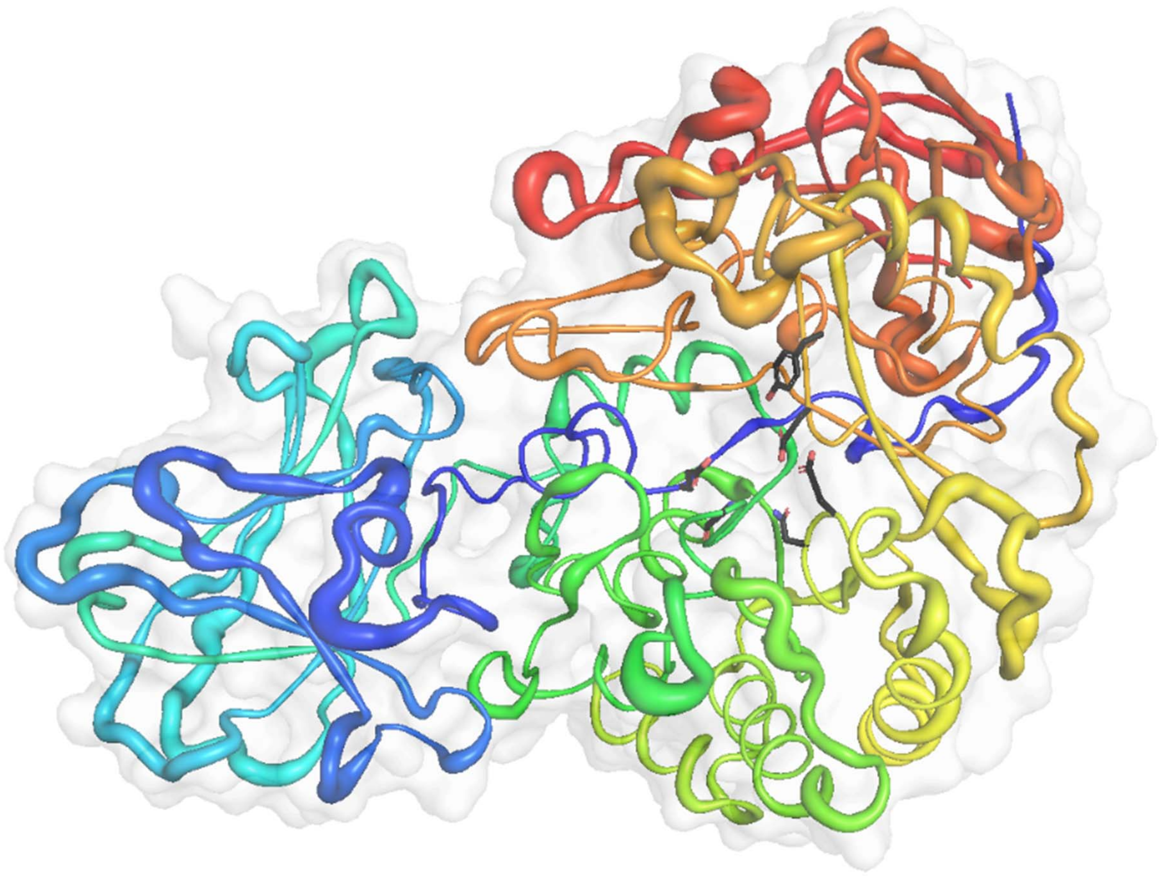

Figure S7 Structure of *Mg*GH51 showing the conservation score determined by ConSurf at each amino acid position as the thickness of the tube representation. A thicker tube indicates higher sequence diversity. The key residues forming the *Mg*GH51 active site are shown as black sticks.
